# Proliferative Activity in Libyan Breast Cancer with Comparison to European and Central African Patients

**DOI:** 10.1155/2013/831714

**Published:** 2013-09-11

**Authors:** Jamela Boder, Fathi Abdalla, Mohamed Elfagieh, Abdelbaset Buhmeida, Yrjö Collan

**Affiliations:** ^1^Department of Pathology, University of Turku, Kiinamyllynkatu 10, 20520 Turku, Finland; ^2^Department of Pathology, National Cancer Institute, Misurata, Libya; ^3^Department of Surgical Oncology, National Cancer Institute, Misurata, Libya; ^4^Center of Excellence in Genomic Medicine Research, King Abdul-Aziz University, P.O. Box 80216, Jeddah 21589, Saudi Arabia

## Abstract

*Background*. We evaluated the relation of proliferative indices with clinicopathological features and prognosis in breast cancer (BC) of Libyan female patients. The data were compared with corresponding results in Finland and Nigeria. *Patients and Methods*. Histological samples of breast cancer from 130 patients were retrospectively studied. Mitotic activity index (MAI) and standardized mitotic index (SMI) were estimated. *Results*. There were statistically significant correlations between the proliferative indices and most clinicopathological features, with the strongest association observed for histological grade (*P* = 0.01 for SMI and *P* = 0.003 for MAI). The proliferative differences between Libyan, Nigerian, and Finnish population were prominent. The mean values of SMI and MAI in Libyan BC patients were 32.1 mitotic figures per square millimeter and 27.3 mitotic figures per 10 high-power fields, respectively. This is clearly lower than those in Nigeria but much higher than those in Finland. The differences between countries are seen in whole material and are also present in subgroups. The results indicated that mitotic activities can be reliable prognostic indicators in Libyan BCs, as they were among Finnish and Nigerian females. Univariate and multivariate analyses found at cut-offs of 19 and 44 mitosis/mm^2^ of SMI were the most significant prognostic factors. *Conclusions*. Proliferative indices with careful estimation of the MAI and SMI could be applied as quantitative criteria for Libyan BC to separate the patients into good, moderate, and bad prognosis groups.

## 1. Introduction 

Mitotic count is the best prognosticator of survival in breast cancer in Caucasian human population, particularly in lymph node-negative patients [1–5]. There is evidence that this might also be true of African breast cancer [6]. However, at population level, African breast cancer differs from European breast cancer in that there are more premenopausal patients than in Europe [7]. We have found evidence that Libyan breast cancer follows the African characteristics [8]. In this study we investigated how this is reflected in the pattern of mitotic counts in Libyan breast cancer.

The current work on proliferative indices were compared with the proliferative indices in Finnish and Nigerian breast cancer patients which were studied previously by Ikpatt et al., 2002 [6] in Nigerian patients and by Kronqvist et al., 1998 [9] in Finnish patients, by the same method.

## 2. Patients and Methods

The study was performed on paraffin-embedded Libyan female breast cancer samples. All cases were diagnosed at the Department of Pathology, African Oncology Institute, Sabratha, Libya, and Tripoli Medical Centre, Tripoli, Libya, during the years 2000–2006. Patients were excluded from this study on the basis of the following exclusion criteria: histopathology was done elsewhere than in the mentioned study centres, patient history and medical files or specimens were not found, the follow-up was less than 3 months, and paraffin blocks were not available for re-cutting. After exclusion of patients 130 patients remained in the study. 115 patients were treated with modified radical mastectomy with axillary clearance. 15 patients were unfit for surgery due to distant metastases; diagnostic biopsies were used in this study. None of the patients had preoperative radiotherapy or any other form of preoperative adjuvant treatment.

A detailed history, clinicopathological features (age, menopausal status, tumor size, stage, and grade, and lymph node status) were collected from patient files (Tables 1 and 2). The mean age at the time of diagnosis was 46.5 (SD ± 13.4) years. 4.6%, 33.8%, 49.2%, and 12.3% of patients were at stages 1, 2, 3, and 4, respectively.

### 2.1. Treatment and Follow-Up

One hundred three (79.2%) patients were treated by modified radical mastectomy and axillary dissection; 9 (6.9%) patients received neoadjuvant chemotherapy with modified radical mastectomy and axillary lymph node dissection. Diagnostic lumpectomy was done in 2 (1.5%) patients and simple mastectomy in 3 (2.3%) patient. No therapeutic surgical intervention was done for 13 (10.0%) patients with metastasis at time of diagnosis (diagnosis with core biopsy). Adjuvant and neoadjuvant chemotherapies with anthracycline were given to 96 (74.4%) patients, while combined chemotherapy of anthracycline and taxans was given to 17 (13.2%) patients. Three patients received chemotherapy of CMF regime (cyclophosphamide, methotrexate, and 5-FU). No chemotherapy was given to 6 (4.7%) patients with early stage, and 7 (5.4%) patients were unfit to receive chemotherapy. Hormonal treatment (tamoxifen) was given to 69 (53.1%) patients with hormone receptor positive. Axillary radiotherapy was given to node-positive patients (*n* = 103). One patient was in first term of pregnancy, and she was treated by MRM and radiotherapy with adjuvant therapy after therapeutic abortion was done.

Patients were followed up until death or the end of the observation period at the mid of July 2007. Some patients were lost from the follow-up. The follow-up data were collected from patient files. Follow-up time ranged from 4 to 78 months. Average follow-up was 32.9 months. Some patients were lost from follow-up. The patients were seen at 3–6-month intervals, and bone isotope scan and chest and abdominopelvic CAT scan were performed every 6–12 months. Breast cancer was recorded as the underlying cause of death for 34 patients. Three cases died of causes unrelated to breast cancer and were not included as events in survival analysis. No autopsies were performed. The survival period was defined as the time from diagnosis either to the time of death or to the date on which the patient was known to be alive.

### 2.2. Histological Methods

The tumor diameter was measured after surgical removal in 3 dimensions, and then biopsy specimens were fixed in buffered formalin (pH 7.3) and embedded in paraffin. Sections of 5 *μ*m thickness were stained with hematoxylin and eosin stain. The histological typing in our study was based on the World Health Organization, Classification of Tumors [10], and grading of tumors was done according to the modified Bloom-Richardson histopathological grading system [11]. There were many guidelines for identifying mitotic figures, and we applied the criteria described by Baak and Oort [12]. Mitotic figures were characterized by an absent nuclear membrane with clear, hairy extension of nuclear material (condensed chromosomes) either clumped (beginning metaphase), in a plane (metaphase/anaphase), or in separate chromosomal aggregates (anaphase/telophase). The basic idea was that at least one chromosomal end was seen in a mitosis [12]. Two parallel clearly separate chromosome clumps were counted as one mitotic figure. The cytoplasm of the mitotic cells was often larger during mitosis than in the resting cells. There were 95 invasive ductal carcinomas (73.1%), 13 invasive lobular carcinomas (10%), 7 mixed ductal and lobular carcinomas (5.4%), 6 medullary carcinomas (4.6%), 3 papillary carcinomas (2.3%), 5 mucinous carcinomas (3.8%), and 1 metaplastic carcinoma (0.8%).

**Figure 1:**
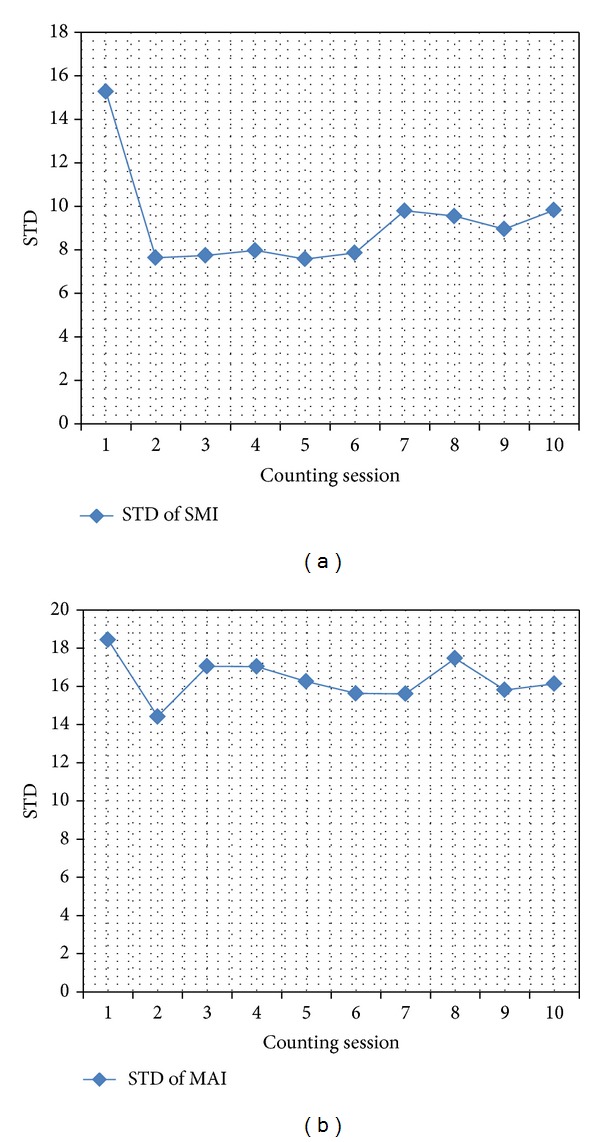
Standard deviations (STD) of the SMI and MAI measured from 10 training samples after 10 training sessions at the beginning of the study (during the first 5 weeks). After the first session STD stabilizes as shown by the curves.

A 5-week training program on mitotic counting was based on set of 10 Libyan female breast cancer samples. During that period counts were repeated on 10 separate occasions, 2-3 days apart. The results of the training phase are shown in Figure 1 [14].

Counting of mitoses was carried out in the most cellular region at tumor periphery, avoiding areas of necrosis, inflammation, in situ carcinoma, and calcification. Since the area of a single HPF may vary from one microscope to another, and this will cause variation in the mitotic score, the standardized mitotic index (SMI) [4, 15, 16] was evaluated in addition to mitotic activity index [9]. 

### 2.3. Estimation of MAI and SMI

We used an Olympus laboratory microscope (objective magnification x40, numerical aperture 0.75 field diameter 490 *μ*m). The number of mitotic figures in 10 consecutive fields from the most cellular area of the sample was the mitotic activity index (MAI). The volume fraction-corrected mitotic index gives the mitotic count as the number of mitotic figures by the area of the neoplastic tissue in the microscopic fields (SMI). This is the number of mitoses in 10 consecutive fields corrected for the volume fraction and field size.

In this method, the area fraction (as estimate of volume fraction) of neoplastic tissue in the microscopic field is evaluated simultaneously with the mitotic count [4, 16]:

(1) $$\begin{array}{c} \text{SMI}=k\left(\frac{\Sigma \text{MI}}{\Sigma Vv}\right), \end{array}$$

where *k* = 100∖*πr* ^2^, *r* is the radius of the microscopic field, in micrometer, and MI = number of mitotic figures in studied field. *Vv* is the volume fraction (estimated by the area fraction, in percent) of the neoplastic tissue in the studied field.

### 2.4. Statistical Analysis

The variables of the material were grouped into logical classes and descriptive statistics calculated for the continuous variables using SPSS 15.0 for Windows. For survival analysis, Kaplan-Meier curves were plotted, and differences between the curves were analyzed using the log-rank test. The MAI and SMI thresholds were the cut-off points showing curve separation with the highest statistical significance. Student *t*-tests and ANOVA were also used to test differences between the groups. In addition, we also performed multivariate survival analysis using Cox's regression model (with classic prognostic predictors entered in a backward stepwise approach with the log-likelihood ratio (L-R) significance test, using the default values for entering and exclusion criteria) to evaluate the independent prognostic value of the studied indices (SMI, MAI, and AI) in addition to MNA and FTD. In all analyses, *P* values below 0.05 were regarded as significant.

## 3. Results

**Figure 2:**
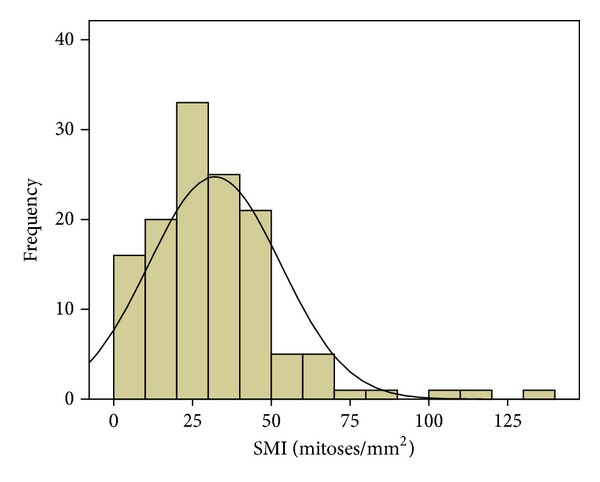
Distribution of standardised mitotic index (SMI) values in 130 Libyan female breast cancers. The mean SMI value was 32. 1 mitoses per square mm.

**Figure 3:**
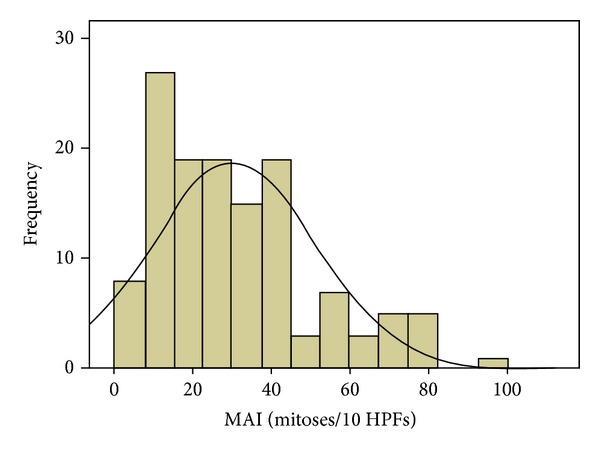
Distribution of mitotic activity index (MAI) values in 130 Libyan female breast cancers. The mean value was 27.3 mitoses per 10 high power-fields.

The clinical characteristics of the Libyan female breast cancer patients (*n* = 130) and average estimates of mitotic indices are described in Tables 1 and 2. The distribution of the values of SMI and MAI is shown in Figures 2 and 3. These distributions are almost but not perfectly similar; 95% of the Libyan female breast cancer patients have the values of SMI between 0 and 67 and values of MAI between 0 and 66.

**Figure 4:**
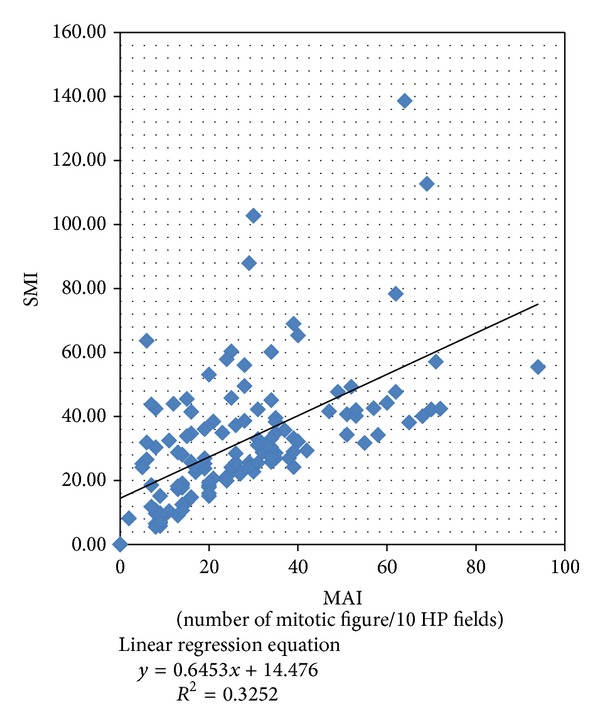
Correlation between SMI (standardized mitotic index) and MAI (mitotic activity index) in 130 Libyan female breast cancers.

The relationship of SMI and MAI is shown in Figure 4. The regression angle between SMI and MAI is about 30° suggesting that on average SMI is smaller than MAI. 

**Figure 5:**
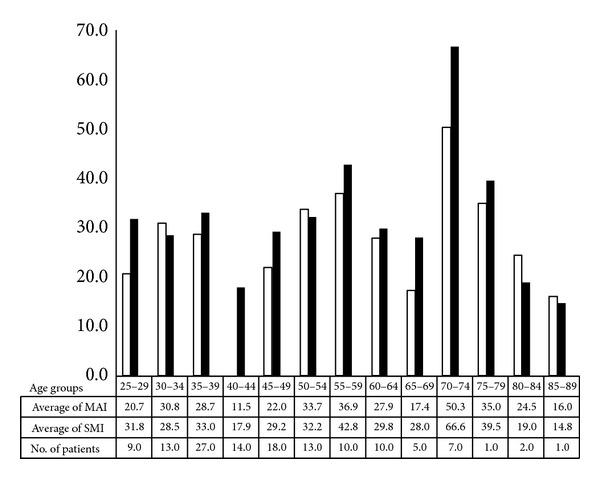
Distributions of mitotic indices through age groups of Libyan female breast cancer patients.

The significances of proliferation indices are shown in Tables 1 and 2. These tables are produced by discerning known clinical characteristics and prognosticators in relevant categories and calculating the averages (±SD) of SMI and MAI in these categories. The highest significance is shown between histological grade and the mitotic counts (*P* = 0.01 for SMI and *P* = 0.003 for MAI). The significances of associations between clinical staging features (clinical stage, TNM stage) are clearer between such features and SMI than between such features and MAI. Age and the menopausal status show a higher significant relationship with MAI (Figure 5). The histological types of the neoplasm do not have a significant relationship with the mitotic counts.

**Figure 6:**
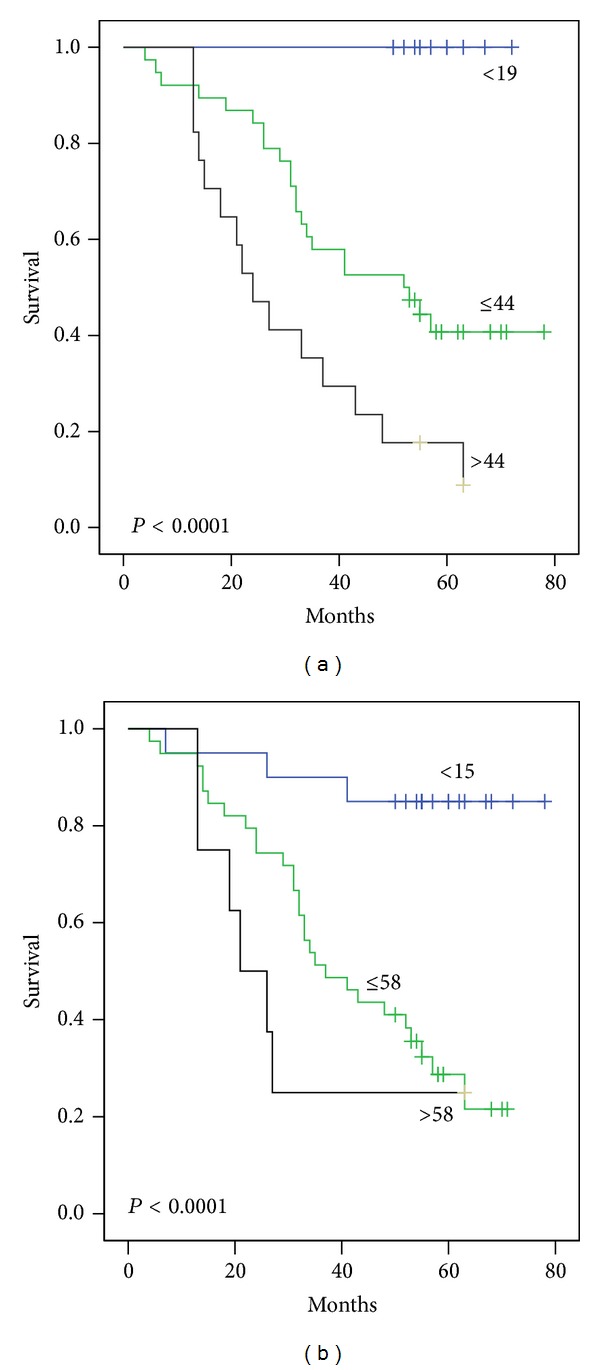
Survival curves for 130 Libyan female patients with breast cancer divided by (a) SMI cut points of 19 and 44 mitoses per square mm. (b) MAI cut points of 15 and 58 mitotic figures/10 hpf. The differences between the curves are very significant.

**Figure 7:**
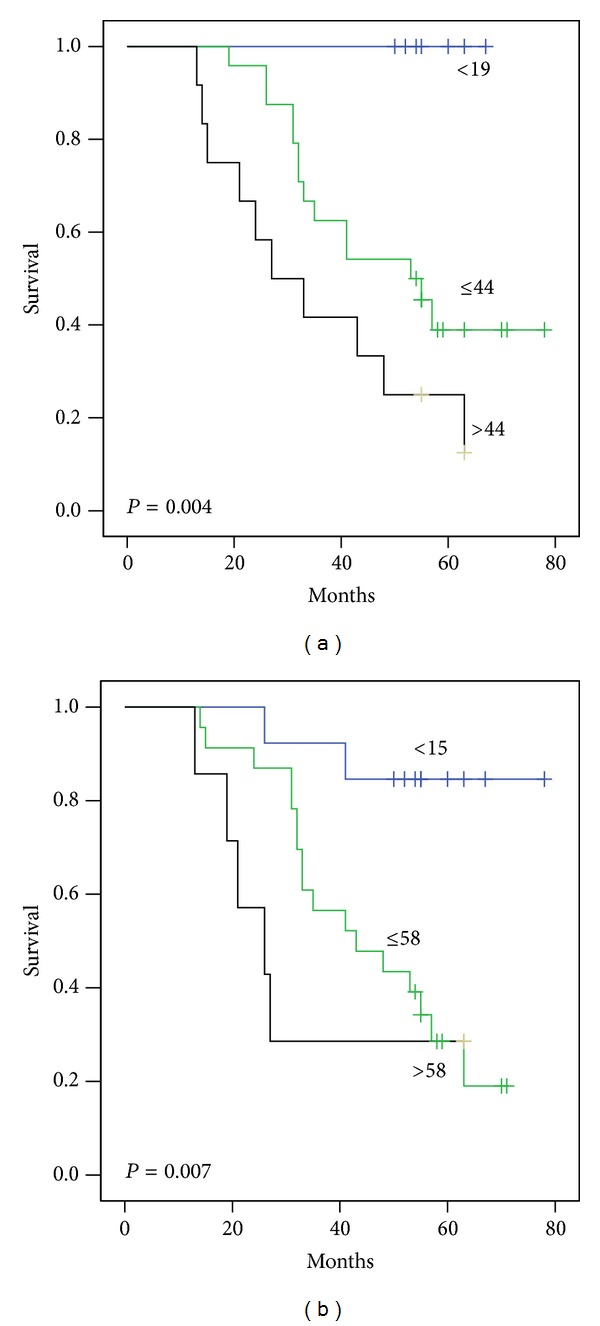
Survival curves for 82 Libyan female patients with IDC of stages 1–3 also divided by (a) SMI cut points of 19 and 44 mitoses per square mm. (b) MAI cut points of 15 and 58 mitotic figures/10 hpf. The differences between the curves are significant.

Univariate (Kaplan-Meier) survival analysis was used to test the value of proliferative indices as a predictor of overall survival; SMI at cut-off 19 and 44 mitoses/mm^2^ was shown to be a significant predictor of overall survival in whole material and IDC with stages 1–3 (*P* < 0.0001 and 0.004, resp.). Similarly MAI at cut-off 15 and 58 mitoses/10 HPF was also significant predictor in whole material and IDC with stage 1–3 (*P* < 0.0001 and 0.007, resp.) (Figures 6 and 7).

### 3.1. Multivariate Cox Analysis

Multivariate analysis of SMI and MAI as continuous and grouping variables was performed according to the Cox model for all breast cancer patients and for IDC with stages 1–3.

To assess the role of SMI as an independent predictor of overall survival, multivariate Cox regression model was used containing the following prognostic predictors: age, stage, menopausal status, nodal status, tumor size, and hormonal status. The multivariate analysis confirmed that higher SMI was an independent factor for poor prognosis (*P* = 0.022), which was independently predicted also by stage (*P* < 0.0001). In a similar analysis entering SMI as grouping variable at cut-off 44 mitoses/mm^2^ confirmed and provided that SMI (*P* = 0.008), age (*P* < 0.02), and clinical stage (*P* < 0.0001) were the independent predictors. 

When the same multivariate analysis model was used to assess the role of MAI as an independent predictor of OAS, the analysis provides that MAI lost its significance as an independent predictor, tumor stage being the only predictor of overall survival (*P* = 0.006). Whereas, in a similar analysis entering MAI as grouping variables at cut-off 58 mitoses/10 HPF provided that clinical stage (*P* < 0.0001) and MAI (*P* = 0.01) were the independent predictors (in grouping variable higher variable groups are used as reference).

Studies of the proliferative indices in Nigerian (Ikpatt et al. [7]) and Finnish patients (Kronqvist et al. [9]) were obtained on invasive ductal carcinoma patients with stages 1,2 and 3 (i.e., without stage 4). Therefore to justify the comparison with these previous studies, we also assess the independent powerful of proliferative indices by multivariate analysis including previous classic prognostic factors after exclude the stage 4 patients from the analysis. SMI as continuous variable and MAI 58 as grouping variable are retained their significance as independent predictors of overall survival with *P* value = 0.04 and 0.05, respectively, in addition to the independent prognosticator of tumor size (*P* < 0.0001).

## 4. Discussion

In addition to the classic estimation of mitosis under microscope and then calculating the MAI and SMI, the proliferation activity can be evaluated by using new immunohistochemistry antibodies directed against different proliferation antigens, such as Ki-67 [17, 18], and proliferating cell nuclear antigen (PCNA) [19], or by analysis of the S-phase fraction using DNA cytometry [17, 20]. However, the S-phase fraction is weak method due to obvious intratumoral heterogeneity [21, 22]. The proliferative indices and immunohistochemistry of Ki-67 index are the most valuable methods. However, Ki-67 has been reported to be more expensive and less independent prognosticator than the proliferative indices [16, 23, 24]. Furthermore, the counting of mitosis may still give more accurate information than the Ki-67. This is might be due to the fact that some of positive Ki-67 cells which are entering in the cell cycle will die before reaching mitosis phase [24]. 

The differences between Central African and European breast cancers have recently emerged [25–30]. The problem that has remained is the characteristics of breast cancer in the Saharan (North African) human population. Libyan breast cancers are optimal for evaluation of this point. It was our intention to study the Libyan breast cancer in respect to proliferation-associated features. Baak et al. [12, 23] found that mitotic count was one the best prognosticator in European breast cancer [18, 19], and later it was shown that, of the two mitotic counting indices (SMI and MAI), the SMI was prognostically stronger than the latter [2, 31]. SMI and MAI are the most actively studied prognostic features.

In the following, we will first deal with differences in SMI and MAI between 3 countries. The proliferative mitotic indices of breast cancer patients in Libya, Finland, and Nigeria are shown in Tables 3 and 4. In Nigerian material the mitotic counts were clearly higher than in our study. On the other hand, in the Finnish material, the results were clearly lower [6, 9]. The proliferative difference in terms of SMI between Libyan and Nigerian tumours and between Libyan and Finnish tumours was significant in the whole material (*P* < 0.0001). However, MAI did not show a significant difference between Libyan and Nigerian tumors. We cannot exclude observer related differences here. The proliferative difference in the lymph node-positive subgroup is clearly significant between Libyan and Finnish and, on the other hand, between Libyan and Nigerian cancers. In lymph node negative subgroups the proliferation measured with SMI was highest among Nigerians and lowest among the Finnish patients. All pairwise relations were statistically significant. That differences between countries are also present in subgroups seems to stress the reliability and consistency of results.

The proliferative difference of the postmenopausal patient groups shows statistically significant difference between Libyan and Finnish patients but does not show such difference between Libyan and Nigerian patients. In fact, Nigerian and Libyan results are near each other. In premenopausal patients, SMI shows statistical significance between Libyan and Nigerian and between Libyan and Finnish tumours, which may reflect biological differences between Central African, North African, and European population, possibly explained by variation in genetic marker distribution in these populations [28, 29].

In contrast with Finnish, the postmenopausal Libyan patients have higher mitotic activity than the perimenopausal patients particularly at age group between 40 and 49 years, whereas there were no significant differences mitotic activity between patients with ages below 40 and above 49 years (*P* = 0.13, *P* = 0.15 in MAI and SMI, resp.). This may be explained by that the peak of oestrogen effect is expected in patients with age below 40, because this period shows high level of serum oestrogen, and the fraction of unmarried female individual was higher among the Libyan breast cancer patients as compared to the North African population (23.8% and 15–21%, resp.) [29]. On other hand, the high mitotic activity of Libyan patients older than 49 years can be explained by diagnosis delay which is a serious problem in Libya [30]. Diagnosis delay was significantly associated with old age and advanced stages [30]. This can be as indicator of advanced disease as high mitotic activity.

Ikpatt et al. [6] also reported higher mean values of proliferative indices in postmenopausal than premenopausal Nigerian patients.

The SMI difference in the grade 3 cancers is significantly larger in Nigerian breast cancer patients than in Libyan patients. This may reflect the fact that Nigerian cancers especially have high proliferative activity. This may be related to the reported obesity of Nigerians [6]. There is corresponding proliferation difference between Libyan and Nigerian patients in stages 2 and 3. Lower number of stage 1 cases in Libya may be related to the diagnosis delay and the absence of screening programs for early detection of breast cancer in Libya [30]. 

Basically results on MAI reflect the same differences between proliferations as reported above, although significant differences between Libyan and Nigerian populations are less obvious.

There were no differences in the methodology between three studies; the proliferative differences between Central African, North African, and European patients were pronounced. It might be there were factors related to differences in the patient materials fixation and preparation. The materials from Nigeria, Libya, and Finland were not fixed with the same carefully controlled fixation. Fixation delay is a common problem in Nigerian material (Ikpatt et al. [6]), and part of Finnish materials was fixed after frozen sections analysis; however, author concluded that at least in her material there was no significant variation in counting of mitosis between both types of specimens [32]. However, we know that the impaction of variation in the fixation time and fixation methods on counting of the mitosis is neither significant nor explain the proliferative difference between varied population [32, 33]. Another important factor is that the screening programs for BC are well established in Finland and other European countries as compared to African countries, which might indicate that the European breast cancers are detected at earlier localized stages and low proliferative activity. On the other hand, it may partly be related to biological difference and variation in genetic marker distribution between Central and North African and European populations [28, 29]. We cannot exclude “African” genomic haplotypes (as contrasted to “European” genomic haplotypes in breast cancer). The new classification of breast cancer according to gene expression analysis has recognized that basal-like breast tumours occur at a significantly higher rate among premenopausal African-American patients compared with postmenopausal of African-American and non-African-American patients [34]. Also our study has shown difference between Libyan and Nigerian subgroups further strengthening our suggestion of biological differences between North and sub-Saharan African population. 

Current and earlier studies [8, 35–37] may suggest biological differences. However, because the studies material had a greater fraction of advanced cases, and may be quite small for final conclusions. That may suggested to be more broad research particular at the molecular level to explore the possible gene mutations or other biological factors that may responsible on the differences in the proliferative indices and the other morphological factors among the countries. In addition, improvement in the health care system and health education in Africa is also important in order to increase women awareness and knowledge with breast cancer.

Our study is in line with studies of Baak and Oort [12], Collan et al. [16], Kronqvist et al. [9], Ikpatt et al. [6], and Jalava et al. [24, 38, 39]; all show that low proliferative indices correlate with better prognosis. LN+ and LN− patients had different prognosticators, and proliferative indices seem to be strong in LN+ patients. The data in Elzagheid et al. [5] was on Caucasians, but also in our study there were no deaths in LN− patients (Table 5).

Our results suggested two significant cut points for the proliferative indices in Libyan material (19 and 44 mitoses/mm^2^ for SMI and 15 and 58 mitoses/10 HPF for MAI) that could separate patients into three subgroups with favourable, intermediate, and unfavourable prognosis (Figure 6). These cut points may be more suitable for the Libyan material than cut points used by Ikpatt et al. in Nigerian material (17 and 92 mitoses/mm^2^ for SMI and 10 and 92 mitoses/10 HPF for MAI) [6] or by Kronqvist et al. in Finnish material (17 and 32 mitoses/mm^2^ for SMI and 13 and 35 mitoses/10 HPF for MAI) [9] or by Buhmeida et al. [13] (Table 3).

Univariate and multivariate Cox's regression analysis showed that SMI, MAI at cut-off 58 and clinical stage were independent predicators for overall survival in whole material. Whereas, in IDC with stages 1–3, the SMI, MAI at cut-off 58, age, and tumor size proved to be the independent predictors, this result was in accordance with several authors results [4, 6, 9, 16, 17, 24], who confirmed that the mitotic indices are independent prognostic markers in breast cancer. Studies of the SMI and MAI in Nigerian [6] and Finnish patients [9] showed that the grouping variable of SMI is a powerful prognosticator in both univariate and multivariate analysis. in contrast to breast cancer patients in Saudi Arabi, neither MAI nor SMI proved to have any value as independent predictors. 

Furthermore, the multivariate analysis in Libyan material suggested that SMI is more useful in assessing prognosis than MAI. This seems to be in line to earlier studies [4, 6, 16, 24]. 

## 5. Conclusion

A positive correlation between the proliferative indices and clinicopathological features was observed, and the SMI showed the strongest correlation with grade and clinical stage. Therefore, in Libyan material for prognostic purposes, proliferative indices can be used as prognostic tools and can show association with more aggressive tumour nature and poor survival.

## Figures and Tables

**Table 1:** Average estimates of mitotic activity in breast cancers (as SMI and MAI) in different subgroups of the 130 Libyan female breast cancer patients.

Group	No. of patients	SMI (SD)	*P* value	MAI (SD)	*P* value
Whole material	130	32.1 (20.9)		27.3 (18.5)	

Age groups			0.02		0.001
<40	49	31.6 (14.0)		27.8 (17.3)	
40–49	32	24.3 (19.2)		17.4 (14.6)	
≥50	49	37.6 (26.0)		33.2 (20.0)	
Menopausal status			0.01		0.008
Premenopausal Postmenopausal	80 50	28.5 (16.6) 37.7 (25.7)		23.9 (17.0) 32.7 (19.8)	
Lymph node (LN) status			0.003		0.035
LN−	27	21.5 (13.0)		20.6 (19.7)	
LN+	103	34.8 (21.8)		29.0 (17.9)	
Histological grade			0.01		0.003
1	10	14.9 (9.2)		12.8 (9.6)	
2	70	32.1 (19.3)		25.4 (17.1)	
3	50	35.4 (23.3)		32.7 (20.0)	
Histological type			0.7		0.8
Invasive ductal carcinoma	95	32.7 (21.2)		26.9 (18.6)	
Invasive lobular carcinoma	13	32.9 (16.4)		30.1 (17.1)	
Other types	22	28.7 (27.7)		27.7 (19.7)	

**Table 2:** Mean estimates (SD) of mitotic activity (SMI, MAI) in different  TNM stages and clinical stages of 130 Libyan female patients with breast cancer. The significance of correlation of stage features and proliferative features is shown as *P*  values evaluated with ANOVA and  *t*-test.

Group	No. of patients	SMI (SD)	*P* value	MAI (SD)	*P* value
*T Stage			0.03		0.06
T1	6	14.0 (11.3)		9.0 (9.2)	
T2	45	28.5 (18.8)		26.6 (18.7)	
T3	44	37.0 (25.4)		27.4 (19.2)	
T4	35	33.5 (16.3)		31.0 (17.3)	
**N stage			0.007		0.02
N0	27	21.5 (13.0)		20.6 (19.7)	
N1	69	34.2 (23.6)		28.6 (18.4)	
N2	32	34.7 (16.7)		28.1 (16.1)	
N3	2	60.1 (25.7)		57.5 (6.4)	
***M stage			0.06		0.1
M0	113	30.8 (20.0)		26.3 (18.4)	
M1	16	41.3 (25.7)		33.9 (18.7)	
Clinical stage			0.002		0.07
1	6	15.7 (9.3)		10.8 (8.1)	
2	44	25.0 (14.5)		26.3 (20.8)	
3	64	36.1 (22.2)		27.8 (16.7)	
4	16	41.3 (21.0)		33.9 (18.7)	

*T stage: extent of the primary neoplasm; **N stage: lymph node status: extent of lymph node involvement; ***M stage: distant metastasis, present/absent.

**Table 3:** Comparison of most significant quantitative threshold values for proliferative indices of Libyan, Nigerian, Finnish, and Saudi Arabian female breast cancer patients.

Country	No. of Patients	SMI mitoses∖mm^2^	MAI mitoses∖10 HPF
Libya	130	19 and 44	15 and 58
Nigeria*	300	17 and 92	10 and 92
Finland°	364	17 and 32	13 and 35
Saudi Arabia^∝^	87	4	13

*Ikpatt et al. 2002 [6], °Kronqvist et al. 1998 [9], ^∝^Buhmeida et al. 2011 [13].

**Table 4:** Proliferative indices of Libya, Nigeria, and Finland in whole patient materials and in subgroups.

		Libya *n* (130)	Finland° *n* (364)	Nigeria* *n* (300)	*P* value	
					Libya versus Finland	Libya versus Nigeria
SMI	Whole material	32.1 (21.0)	13.8 (17.8)	42.6 (27.5)	<0.0001	<0.0001
MAI		27.3 (18.5)	10.7 (16.5)	30.5 (25.1)	<0.0001	0.192

SMI	LN+	34.8 (21.8) *n* = 103	17.8 (19.3) n = 131	45.4 (27.6) *n* = 235	<0.0001	0.001
	LN−	21.5 (13.0) n = 27	11.6 (16.5) *n* = 232	32.6 (25.1) *n* = 65	0.003	0.032
	Postmenopause	37.7 (25.7) n = 50	11.2 (13.4) n = 249	44.9 (26.6) *n* = 77	<0.0001	0.134
	Premenopause	28.5 (16.6) *n* = 80	19.6 (29.0) n = 114	41.9 (27.8) *n* = 223	0.014	<0.0001
	Grade 1	14.9 (9.2) *n* = 10	NA	11.8 (11.4) *n* = 44	NA	0.427
	Grade 2	32.1 (19.3) n = 70	NA	31.7 (16.5) *n* = 119	NA	0.880
	Grade 3	35.4 (23.3) *n* = 50	NA	61.9 (24.5) *n* = 137	NA	<0.0001
	Stage 1	15.7 (9.3) *n* = 6	NA	32.6 (25.1) *n* = 65	NA	0.108
	Stage 2	25.0 (14.5) *n* = 44	NA	41.9 (28.3) *n* = 75	NA	<0.0001
	Stage 3	36.1 (22.2) *n* = 64	NA	48.9 (28.8) *n* = 98	NA	0.003
	Stage 4	41.3 (21.0) *n* = 16	NA	44.3 (24.4) *n* = 72	NA	0.650

MAI	LN+	29.0 (17.9) *n* = 103	13.4 (17.4) n = 131	32.7 (25.6) *n* = 235	<0.0001	0.184
	LN−	20.6 (19.7) *n* = 27	9.2 (15.8) *n* = 232	22.6 (21.2) *n* = 65	0.001	0.675
	Postmenopause	32.7 (19.8) *n* = 50	8.8 (11.9) *n* = 249	33.8 (24.9) *n* = 77	<0.0001	0.793
	Premenopause	23.9 (17.0) *n* = 80	14.9 (23.2) *n* = 114	29.4 (22.1) *n* = 223	0.004	0.044
	Grade 1	12.8 (9.6) *n* = 10	NA	4.2 (3.8) *n* = 44	NA	<0.0001
	Grade 2	25.4 (17.1) n = 70	NA	16.8 (10.1) *n* = 119	NA	<0.0001
	Grade 3	32.7 (20.0) n = 50	NA	50.9 (21.9) *n* = 137	NA	<0.0001
	Stage 1	15.7 (9.3) n = 6	NA	22.6 (21.2) *n* = 65	NA	0.434
	Stage 2	25.0 (14.5) *n* = 44	NA	28.3 (24.4) *n* = 75	NA	0.416
	Stage 3	36.1 (22.2) n = 64	NA	36.6 (26.8) *n* = 98	NA	0.901
	Stage 4	41.3 (21.0) *n* = 16	NA	31.9 (24.7) *n* = 72	NA	0.162

Data on Nigeria and Finland are basically the same as those published in the study of *Ikpatt et al. [6, 7] and °Kronqvist et al. [9]. Significance is estimated by one-way ANOVA test.

**Table 5:** Univariate analysis on the significance of the most important prognosticators in the whole Libyan material using cut points at 19 and 44 mitotic figures/mm² and 15 and 58 mitotic figures/10 hpfs for SMI and MAI, respectively. Different subgroups of the whole material are also tested in the corresponding method.

Group of patients	Prognostic feature	*P* value
All patients	SMI 19	0.0001
	SMI 44	0.001
	MAI 15	0.0001
	MAI 58	0.1
	LN status	<0.0001
	T stage	<0.0001
	M stage	<0.0001
	Grade	0.003

Premenopausal	SMI 19	0.005
	SMI 44	<0.0001
	MAI 15	<0.0001
	MAI 58	0.13
	LN status	<0.0001
	T stage	<0.0001
	M stage	0.027
	Grade	0.002

Postmenopausal	SMI 19	0.1
	SMI 44	0.09
	MAI 15	0.8
	MAI 58	0.5
	LN status	0.1
	T stage	0.06
	M stage	<0.0001
	Grade	0.7

Positive LN	SMI 19	0.009
	SMI 44	0.006
	MAI 15	0.02
	MAI 58	0.06
	T stage	0.001
	M stage	<0.0001
	Grade	0.09

Negative LN	SMI 19	0.4
	SMI 44	0.7
	MAI 15	0.1
	MAI 58	0.7
	T stage	0.7
	M stage	NA
	Grade	0.7
